# Efficacy and Safety of Agomelatine in Depressed Patients with Diabetes: A Systematic Review and Meta-Analysis

**DOI:** 10.3390/ijms252312631

**Published:** 2024-11-25

**Authors:** Adam Gędek, Szymon Modrzejewski, Michał Materna, Zofia Szular, Adam Wichniak, Paweł Mierzejewski, Monika Dominiak

**Affiliations:** 1 Department of Pharmacology, Institute of Psychiatry and Neurology, 02-957 Warsaw, Poland; 2 Third Department of Psychiatry, Institute of Psychiatry and Neurology, 02-957 Warsaw, Poland; 3 Faculty of Medicine, Medical University of Lublin, 20-059 Lublin, Poland; 4 Babinski Clinical Hospital, 30-393 Krakow, Poland; 5 Faculty of Medicine, Medical University of Warsaw, 02-091 Warsaw, Poland

**Keywords:** agomelatine, depression, diabetes, glycemia, HbA1C, glycemic control, comorbid

## Abstract

Major depressive disorder (MDD) and diabetes mellitus (DM) remain among the most prevalent diseases and the most significant challenges faced by medicine in the 21st century. The frequent co-occurrence and bidirectional relationship between the two conditions necessitates the identification of treatment strategies that benefit both. The purpose of this study was to systematically review and meta-analyze data on the efficacy and safety of agomelatine (AGO) in the treatment of patients with depression with comorbid diabetes to explore its potential mechanism of action in both diseases and its impact on diabetic parameters. Following PRISMA guidelines, a total of 11 studies were identified, both preclinical and clinical trials. Agomelatine has shown great potential as a treatment option for patients with diabetes and comorbid depression and anxiety. In addition to improving depressive and anxiety symptoms, it is also beneficial in glycemic control. A meta-analysis demonstrated a statistically significant reduction in glycated hemoglobin (HbA1C) and fasting blood glucose (FBG) levels following AGO administration over a period of 8–16 weeks. The administration of agomelatine was found to result in a significantly greater reduction in HbA1C than that observed with the selective serotonin reuptake inhibitor (SSRI) medications (namely fluoxetine, sertraline, and paroxetine) during 12–16 weeks of therapy. Furthermore, AGO has been found to be at least as effective as SSRIs in reducing depressive symptoms and more effective than SSRIs in reducing anxiety symptoms. The safety of such treatment is similar to SSRIs; no severe adverse events were reported, and the incidence of some side effects, such as insomnia and sexual dysfunction, are even less often reported. Particularly promising is also its potential action in improving some diabetic complications reported in preclinical trials. This might be through mechanisms involving the reduction in oxidative stress, anti-inflammatory effects, and potentially noradrenergic or NMDA receptor modulation. Further clinical studies on larger sample sizes, as well as elucidating its mechanisms of action, especially in the context of diabetic complications, are needed. Research should also focus on identifying the patient subpopulations most likely to benefit from agomelatine treatment.

## 1. Introduction

Diabetes mellitus is a common metabolic disorder characterized by impairments in insulin secretion or action. It is estimated that more than 520 million people worldwide are affected by this condition [1]. The most prevalent form of diabetes is type 2 diabetes mellitus (T2DM), which frequently co-occurs with depression and anxiety disorders [2,3]. Major depression is a common mental disorder that markedly diminishes the quality of life and reduces life expectancy [4,5]. It affects approximately 250 million people worldwide [6]. It is hypothesized that both diabetes and depression may share a common etiology, with evidence indicating a bidirectional relationship between the two conditions. This suggests that both disorders can affect and exacerbate each other’s progression [7,8,9]. Depression can affect food preferences, food intake, and physical activity, changing energy balance, which may manifest as overweight and a reduction in self-care activities [10,11]. Additionally, depression can stimulate the hypothalamic–pituitary–adrenal hormonal axis, leading to elevated cortisol levels and increased levels of inflammatory cytokines, which may contribute to the development of diabetes [12,13]. Conversely, the implementation of measures designed to enhance the management of diabetes, such as dietary restrictions and the promotion of social and physical activities, has been observed to improve depressed mood [14]. Furthermore, patients with depression and diabetes have an elevated mortality rate, an increased risk of developing complications associated with diabetes, a higher likelihood of requiring hospitalization, and a reduced quality of life [15,16,17,18]. Diabetes can result in several acute complications, including hypoglycemic and lactic acidosis coma, diabetic ketoacidosis, and hyperosmolar non-ketotic syndrome [19]. Furthermore, long-term hypoglycemia can cause chronic complications, including retinopathy, nephropathy, neuropathy, cardiovascular issues, and diabetic encephalopathy [20,21,22,23]. These conditions collectively contribute to patients’ suffering, diminished quality of life, and premature mortality, thus positioning diabetes as a significant challenge of the 21st century.

The management of both depression and diabetes continues to present a significant challenge for clinicians. The results of previous studies indicate that different classes of antidepressants contribute to weight gain by affecting metabolic processes and increasing food intake [24]. This applies to the first-line pharmacological treatment of depression—selective serotonin reuptake inhibitors (SSRIs)—as shown by the long-term observational studies [25]. However, studies have indicated that they may have a beneficial impact on the management of diabetic parameters [26]. A recent meta-analysis of randomized clinical trials demonstrated that melatonin supplementation also exerts a beneficial influence on diabetic parameters [27]. Furthermore, it has been demonstrated to alleviate complications associated with the condition [28]. This indicates that the melatonin MT1 and MT2 receptors agonist and also serotonin 5-HT2C receptor antagonist agomelatine (AGO) may be a promising alternative treatment for comorbid depression and diabetes, along with its complications, as evidenced by the initial reports on this subject [29,30].

The objective of this systematic review was to synthesize the existing preclinical and clinical evidence on the effects of agomelatine (AGO) on diabetes and its complications. The specific objectives were as follows: (1) to assess the impact of AGO administration on diabetic parameters; (2) to compare AGO versus SSRI administration on diabetic parameters; (3) to assess the influence of AGO on depressive and anxiety symptoms in patients with diabetes; (4) to evaluate the safety profile of AGO; and (5) to evaluate the potential molecular mechanism of AGO action.

## 2. Methods

This systematic review was conducted according to the PRISMA statement. Two reviewers independently conducted each stage throughout the review process based on the previously prepared protocol. Any disagreements were resolved via discussion and the opinion of the senior researcher.

### 2.1. Eligibility Criteria

The PICO model was adapted for preclinical, observational, and interventional trials (Table 1). Each relevant publication was assessed based on predefined eligibility criteria and categorized as preclinical or clinical (observational or interventional).

The inclusion criteria were as follows: (1) preclinical or clinical study of any design; (2) study using agomelatine on patients with diabetes or animal models; (3) age over 18 (applies to clinical studies); (4) published in English or Polish. The exclusion criteria were as follows: (1) lack of compatibility with PICO; (2) research using cell lines; (3) full-text was not available; (4) not an original article; (5) not published in a peer-reviewed journal; and (6) reviews, systematic reviews, meta-analysis, and pooled analysis.

### 2.2. Search Strategy

In March 2023, PubMed, Web of Science, and Scopus were systematically searched using the keywords “agomelatine” and “diabetes” by two independent reviewers. Additionally, clinicaltrial.gov register search and follow-up citation scanning were also performed. Results were downloaded into Mendeley Desktop, and two investigators independently identified potentially relevant articles, removed duplicates, and then reviewed titles and abstracts. The studies were then evaluated for eligibility criteria. The researcher’s reliability level was assessed with Cohen’s kappa coefficient (κ), which equaled κ = 0.97. All uncertainties were resolved through discussion and consultation with the senior researcher.

### 2.3. Data Extraction

Data related to the effects of agomelatine on diabetes were extracted independently using a tailored form. The form included authors, year of publication, country, sample and control size, duration, characteristics of the research and control group (sex, mean age, diagnosis, and treatment), dose of agomelatine, and outcomes.

### 2.4. Quality Assessment

The risk of bias in the clinical trials was evaluated in accordance with the guidelines set forth by the Cochrane Collaboration [31]. The Rob2 (Revised Cochrane risk of bias tool for randomized trials; current version, 22 August 2019 version) was used to evaluate the clinical trials in accordance with the following domains: randomization process, deviation from intended intervention, missing outcome data, measurement of the outcome, and selection of the reported results [32]. The risk of bias assessment was conducted for each study individually, with the results presented separately for each study and cumulatively for each domain. The Robvis tool was employed for visualization purposes [33]. At least two authors independently assessed the paper, establishing consensus when disagreements arose.

### 2.5. Synthesis and Analysis

In a predefined study protocol, we created a PICO model for preclinical, observational, and interventional studies. However, we did not identify any observational studies, so further analysis is presented separately in two groups for preclinical and clinical—interventional studies. For clinical studies meta-analysis was available to conduct. Continuous outcomes were pooled as standardized mean difference (SMD). I^2^ statistic was used to estimate the heterogeneity of the studies, and a *p*-value of 0.05 was used as the cut-off point for significance. If heterogeneity was present, a random effects model was used to calculate the standardized mean difference. I^2^ values of 25, 50, and 75 were considered to indicate mild, moderate, and substantial heterogeneity.

## 3. Results

### 3.1. Study Selection

**Figure 1:**
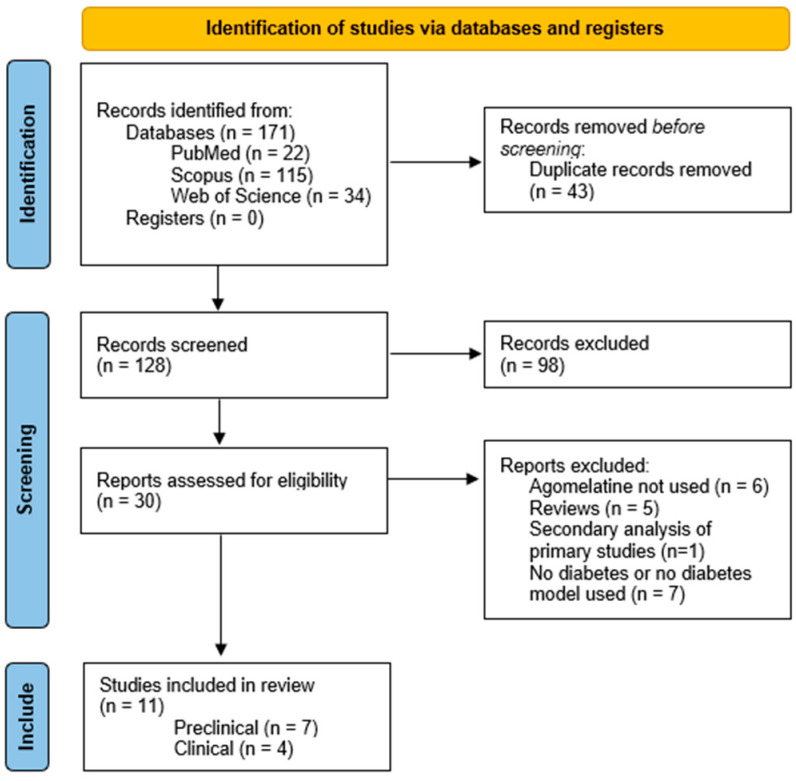
Flow chart: study selection process.

A total of 171 articles were identified through the search strategy. After the removal of 43 duplicates, 128 remained for titles and abstract screening. The initial assessment of 30 full-text articles for eligibility resulted in a reduction to 11 articles that met the inclusion criteria. The search process flow and results are detailed in Figure 1.

### 3.2. Description of Studies

The included studies were published between the years 2013 and 2023. Among them, seven were preclinical [29,34,35,36,37,38,39], and four were clinical trials [30,40,41,42]. All preclinical studies involved rodents and used streptozotocin-induced diabetes models. In addition to assessing the effects on diabetic parameters, they also evaluated the efficacy of agomelatine in diabetic complications such as neuropathy [29,34,37], encephalopathy and cognitive impairment [35,36], nephropathy [39], and dysfunction of the male reproductive system [38]. Regarding clinical trials, one was a single-blind randomized controlled trial (RCT) [41], one was a double-blind RCT [42], and one was a randomized open-label study [40]. One clinical trial, although described as observational by authors, was classified as an interventional study because of the random assignment of participants to two pharmacological treatment arms [30]. One excluded study [43] was a secondary analysis of two included interventional studies [41,42]. The total patient sample was 280, and the average study time was 12 weeks (range of 8–12 weeks). The majority of studies were conducted in Turkey (5) and China (2), with single trials from France (1), Egypt (1), India (1), and Greece (1).

### 3.3. Quality Assessment of Included Studies

**Figure 2:**
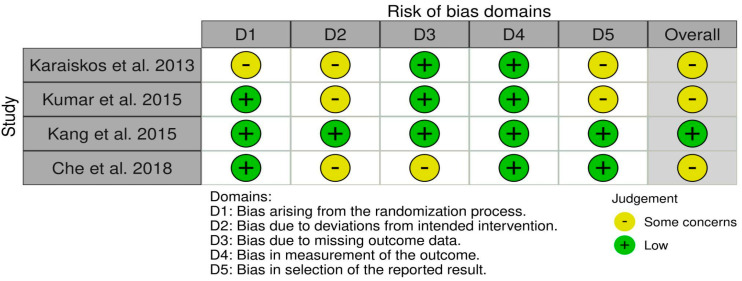
Risk of bias for clinical studies with RoB2 tool (current version; 22 August 2019 version) [30,40,41,42].

**Figure 3:**
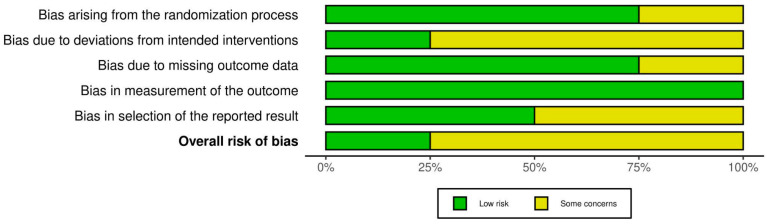
Risk of bias summary for clinical studies with RoB2 tool.

The primary clinical studies were evaluated according to the RoB2 tool. The risk of bias for each individual study is presented in Figure 2, while the summary assessment is shown in Figure 3.

### 3.4. Preclinical Studies

A total of seven preclinical studies using animal models were identified (Table S1—Supplementary Materials). The studies evaluated the efficacy of agomelatine in models of diabetes complications in rats, including neuropathy [29,34,36,37], nephropathy [39], cognitive impairment [35], and dysfunction of the male reproductive system [38].

#### 3.4.1. Effect of Agomelatine on Diabetic Parameters Measured in Blood

Some of the preclinical studies have demonstrated a favorable impact of agomelatine on diabetic parameters; however, the summary results are inconclusive. Two studies reported a reduction in blood glucose levels following agomelatine administration (2/4, 50%), an increase in serum insulin levels (2/2, 100%) [36,39]. In one study, the group taking agomelatine exhibited a lower HOMA-IR (1/1, 100%) [39]. However, these results were inconclusive, as two studies demonstrated no difference in blood glucose (2/4, 50%) [29,38] and one in OGTT (1/1, 100%) between groups [29]. The administration of agomelatine did not result in any discernible impact on metabolic parameters, body weight [29], or the majority of lipid parameters [36].

#### 3.4.2. Effect of Agomelatine on Models of Diabetic Complications in Rats (Nephropathy, Neuropathy, Reproductive System Damage, Encephalopathy and Cognitive Impairment) and Potential Mechanisms

The results of the three studies (3/3, 100%) indicated that agomelatine had a beneficial effect on diabetic neuropathy [29,34,37]. AGO was observed to significantly improve mechanical and thermal hyperalgesia and allodynia responses [29], reduce hypersensitivity [34], and increase the pain threshold [37] in diabetic rats. In the studies, a positive effect was observed following the co-administration of agomelatine with gabapentin [34] or morphine [37]. In addition to the significant effects on melatoninergic and 5HT2C receptors [34], one study indicated that both alpha and beta adrenoceptors contributed to the pharmacological action of AGO on neuropathic pain [29], while another demonstrated that alpha-2 adrenoceptors, but not beta adrenoceptors, were involved in this process [34]. Furthermore, the concomitant administration of morphine with AGO resulted in a notable reduction in the elevation of NMDA type subunit-1 (GluN1) mRNA levels in the raphe nucleus and a considerable attenuation of the increase in GluN1 mRNA levels in the PAG when compared to the levels observed in the morphine-alone group [37].

Two studies reported the positive effects of AGO on diabetic encephalopathy or cognitive impairment (2/2, 100%) and demonstrated that treatment effectively reversed the impaired learning, memory performance, and emotional learning of diabetic rats with comparable efficacy to piracetam [35]. AGO pretreatment reversed the diabetes-induced reduction in cells in the CA1–3 regions and decreased the dentate gyrus [35]. Additionally, AGO was observed to reverse the reduction in cell viability induced by high glucose in DRG neurons in a neuropathic pain model [36]. Ozcan et al.’s study emphasized the role of central anti-inflammatory changes induced by agomelatine as a potential mechanism of improvement in encephalopathy, increasing total antioxidant capacity, reducing IL-1β mRNA levels in the nucleus accumbens and raphe nucleus, as well as TACR1 mRNA levels in the raphe nucleus, PAG, amygdala, and nucleus accumbens [36].

A single study demonstrated a favorable impact of AGO on diabetic nephropathy (1/1, 100%). The administration of AGO was observed to improve kidney function, as indicated by serum creatinine, urea, and KIM-1 levels, as well as morphology, as indicated by a marked reduction in interstitial fibrosis and tubular epithelial degeneration. AGO treatment, presumably via melatonin receptors, elevated SIRT1 expression, diminished renal NFκB phosphorylation, augmented renal AMPK phosphorylation, and mitigated renal inflammation (decreased levels of ICAM-1, VCAM-1, and MCP-1) [39].

One study demonstrated that AGO exerts a beneficial effect on testicular damage induced by diabetes (1/1, 100%). The loss of somatic sertoli cells and spermatogenic series cells was significantly reduced in the AGO group in comparison to the diabetic group that did not receive treatment. The potential mechanism may be linked to oxidative stress processes, as evidenced by decreased MDA levels and increased CAT and SOD enzyme levels, and inflammation, as evidenced by decreased expression of TNFα, NOS2, fibronectin, VEGF, and TUNEL-positive cells in the AGO group compared to the diabetic rats without treatment [38].

### 3.5. Clinical Studies

Four clinical trials were included for analysis in this systematic review (Table 2) [30,40,41,42]. All four studies compared the efficacy and safety of agomelatine with SSRI drugs—fluoxetine [41], paroxetine [42], sertraline [30], or escitalopram [40]. Agomelatine was used at a dose of 25 to 50 mg/d.

#### 3.5.1. Efficacy of Agomelatine on Diabetic Parameters

**Figure 4:**
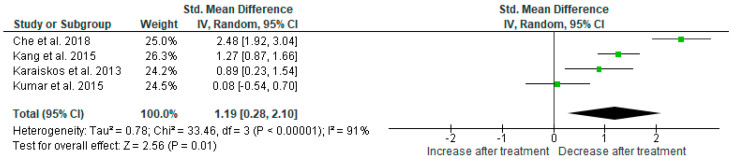
Meta-analysis on the effect of agomelatine on glycated hemoglobin [30,40,41,42].

Results from four studies (*n* = 144) were included in the meta-analysis on the effect of AGO on glycated hemoglobin (HbA1C) (Figure 4). Using the random-effects model, we found a significant HbA1C level reduction after AGO administration (*p* = 0.01). Heterogeneity among studies was high (I^2^ = 91%). Sensitivity analysis did not show any change in the result after the exclusion of each individual study.

**Figure 5:**
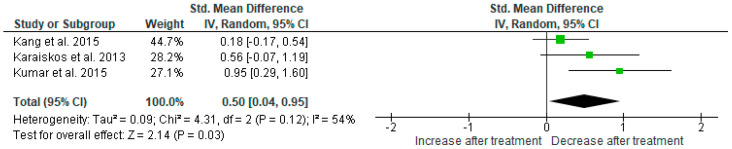
Meta-analysis on the effect of agomelatine on fasting blood glucose [30,40,42].

Results from three studies (*n* = 100) were included in the meta-analysis on the effect of AGO on mean fasting blood glucose (FBG) (Figure 5). Using the random-effects model we did find a significant FBG level reduction after AGO administration (*p* = 0.01). Heterogeneity among studies was moderate (I^2^ = 54%). Sensitivity analysis did not show any change in the result after the exclusion of each individual study.

#### 3.5.2. Efficacy of Agomelatine on Diabetic Parameters Versus Other Antidepressive Treatment

**Figure 6:**
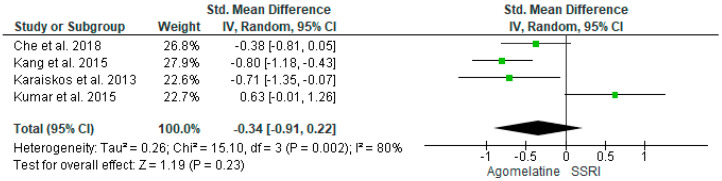
Meta-analysis comparing the effects of agomelatine and SSRIs on glycated hemoglobin, including the outlier [30,40,41,42].

**Figure 7:**
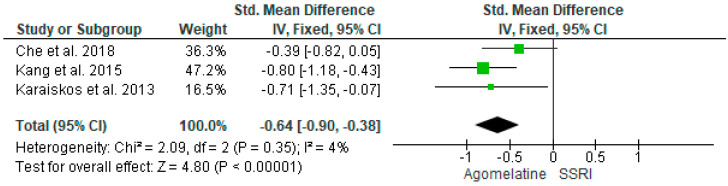
Meta-analysis comparing the effects of agomelatine and SSRIs on glycated hemoglobin, not including the outlier [30,41,42].

In three out of four studies (3/4, 75%), agomelatine 25–50 mg/d significantly lowered glycated hemoglobin at the endpoint (12–16 weeks) than SSRIs [30,41,42]. In one study, escitalopram was more effective than agomelatine 25 mg/d (8 weeks) [40]. Results from four studies (*n* = 280) were included in the meta-analysis on the effect of AGO on HbA1C versus other antidepressive treatments. Using the random-effects model, we did not find a significantly greater HbA1C level reduction in the AGO group than SSRIs (*p* = 0.23). Heterogeneity among studies was high (I^2^ = 80%) (Figure 6). However, after exclusion, the main heterogeneity contributing study [40] AGO significantly better reduced HbA1C than other SSRIs (*p* < 0.00001, I^2^ = 4%), also using random and fixed models (Figure 7).

**Figure 8:**
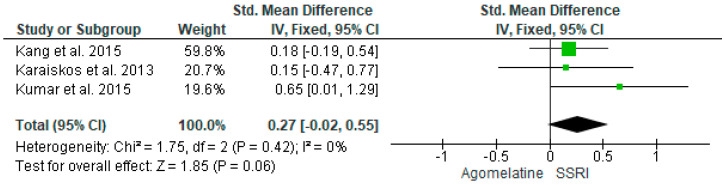
Meta-analysis comparing the effects of agomelatine and SSRIs on fasting blood glucose [30,40,42].

In one study, escitalopram significantly reduced fasting blood glucose compared to agomelatine [40], while there was no difference between agomelatine compared to paroxetine, fluoxetine, and sertraline [30,41,42]. Results from 3 studies (*n* = 196) were included in the meta-analysis on the effect of AGO on FBG versus other antidepressive treatments. Using the fixed-effects model, we did not find a significantly greater FBG level reduction in the AGO group than SSRIs; however, a trend towards significance has been observed (*p* = 0.06). Heterogeneity among studies was low (I^2^ = 0%) (Figure 8).

#### 3.5.3. Efficacy of Agomelatine on Psychiatric Symptoms Versus Other Antidepressive Treatment

In one of the four clinical studies (1/4, 25%), the agomelatine-treated group had a significantly lower Hamilton Depression Rating Scale (HDRS) score after 16 weeks of treatment [30]. In two other studies (2/4, 50%), the HDRS difference was not significant, although the averages were lower in the AGO group after 12 weeks of treatment [41,42]. Similar results were collected in the context of remitters and responders. These studies used an agomelatine dose of 25–50 mg/d. According to one study (1/4, 25%), HDRS and Montgomery–Åsberg Depression Rating Scale (MADRS) scores of the escitalopram group were significantly lower than AGO group at 4 and 8 weeks [40]. This study used 25 mg/d of agomelatine.

Three studies evaluated the effect of agomelatine on anxiety symptoms in patients with diabetes. In each of the three studies (3/3, 100%), agomelatine significantly reduced the Hamilton Anxiety Rating Scale (HARS) score compared to SSRI drugs (paroxetine, fluoxetine, sertraline) at the endpoint [30,41,42]. In all studies, the dose of the drug was 25–50 mg/d. Regarding the time effect, in the study by Kang et al. 2015, the difference was not significant at week 6 and gained significance after 12 weeks of treatment [42]. According to Che et al. 2018, it was not significant at week 4 but became significant at weeks 8 and 12 [41]. HARS was also significantly lower after 16 weeks of treatment, according to Karaiskos et al. 2013 [30].

#### 3.5.4. Safety of Agomelatine Versus Other Antidepressive Treatment

There was no difference between patients who dropped out of the study between the agomelatine group and other SSRI groups [30,40,41,42]. Two studies reported fewer side effects in the group receiving agomelatine [30,41]. No study reported significant side effects as a complication of taking the drug for up to 16 weeks. No study reported an increase in liver enzymes, which should be monitored during agomelatine treatment.

**Table 2:** Clinical studies—data summary.

Authors, Year, Country	Duration, Design	Sample Size, Population, Age Range, Sex F/M, Antidiabetic Treatment	Treatment	Results: (1) Psychiatric Symptoms; (2) Diabetic Parameters; (3) Safety
Che et al., 2018 [41] China	12 weeks, single-blind RCT	*n* = 84 Patients with T2DM (HbA1C > 7%), depression (≥17 HDRS), and anxiety (>7 HARS); Aged 18–80 Sex 48/36 It was not controlled which antidiabetic drugs and in what doses patients receive.	Agomelatine 25–50 mg/d Fluoxetine 30–40 mg/d	(1) Compared with the fluoxetine group, the AGO group had a significantly lower average HARS score at weeks 8 and 12. HDRS score, response, and remission rates did not differ significantly between groups at the endpoint. (2) Both drugs had no significant effects on BMI and FBG. HbA1c level was significantly decreased in both groups, but was significantly lower in the AGO group at the endpoint. (3) No significant difference in drop-out was observed between the two groups. The incidence of adverse events was significantly lower in the AGO group.
Kang et al., 2015 [42] China	12 weeks, double-blind RCT	*n* = 116 Patients with T2DM (HbA1C > 7%) and depression (DSM-IV, ≥17 HDRS) Aged 27–73 Sex 53/63 Patients received standard antidiabetic treatment.	Agomelatine 30–50 mg/d Paroxetine 20–40 mg/d	(1) At week 12, the HARS score in the AGO group was significantly lower compared with the paroxetine group. The number of remitters and responders was similar in both groups at week 12. (2) The average level of HbA1c was significantly lower in the AGO group compared with the paroxetine group. There was no significant difference in post-treatment HbA1c levels between responders and non-responders. There were no statistically significant differences between the two groups in FBG and BMI, and the effect of treatment on these values was not significant. (3) All of the patients completed the trial. There was no significant difference in adverse events (such as nausea, headache, dry mouth, diarrhea, dizziness, anxiety, insomnia) between the paroxetine group and the AGO group.
Kumar et al., 2015 [40] India	8 weeks, open-label RCT	*n* = 40 Patients with T2DM (ADA 2013, HbA1C > 6.5%) and depression (ICD-10, ≥14 HDRS) Aged >18 Sex no info	Agomelatine 25 mg/d Escitalopram 10 mg/d	(1) HDRS and MADRS scores of the escitalopram group were significantly lower than the AGO group at 4 and 8 weeks. However, the number of study participants was insufficient to infer the efficacy or ineffectiveness of antidepressants using the above clinical scales. (2) The Escitalopram group had a significant reduction in FBG and HbA1C values as compared to the AGO group at 8 weeks. (3) No significant difference in drop-out was observed between the two groups.
Karaiskos et al., 2013 [30] Greece	16 weeks, open-label study	*n* = 40 Patients with T2DM (HbA1C > 7.5%) and depression (DSM-IV) Aged 18–60 Sex no info	Agomelatine 25–50 mg/d Sertraline 50–100 mg/d	(1) HARS and HDRS scores were significantly lower in the AGO group compared to the sertraline group at the endpoint. However, the number of study participants was insufficient to infer the efficacy or ineffectiveness of antidepressants using the above clinical scales. (2) The effect of treatment on the FBG and body weight was not significant. HgA1C was significantly lower in the AGO group compared with the sertraline group at the endpoint. SCI-R score was significantly higher in the AGO group at the endpoint. (3) All of the patients completed the study. More adverse effects were reported in the sertraline group (such as nausea, headache, dry mouth, diarrhea, insomnia). For the AGO group, dizziness and somnolence were reported.

AGO—agomelatine, FBG—fasting blood glucose, HDRS—Hamilton Depression Rating Scale, HARS—Hamilton Anxiety Rating Scale, SCI-R—Self-Care Inventory-Revised, HBA1C—glycated hemoglobin, BMI—body mass index, T2DM—Type 2 Diabetes Mellitus, MADRS—Montgomery–Åsberg Depression Rating Scale, ADA—American Diabetes Association 2013 criteria.

## 4. Discussion

Diabetes frequently co-occurs with depression and anxiety symptoms, representing a significant therapeutic challenge. A recent meta-analysis demonstrated that antidepressants, namely SSRIs, were found to be effective in both reducing depression and improving diabetes control [44]. Further research has provided the premise that the novel melatoninergic antidepressant—agomelatine may have similar effectiveness as SSRIs in managing depression, as well as in controlling diabetic parameters [45,46].

**Figure 9:**
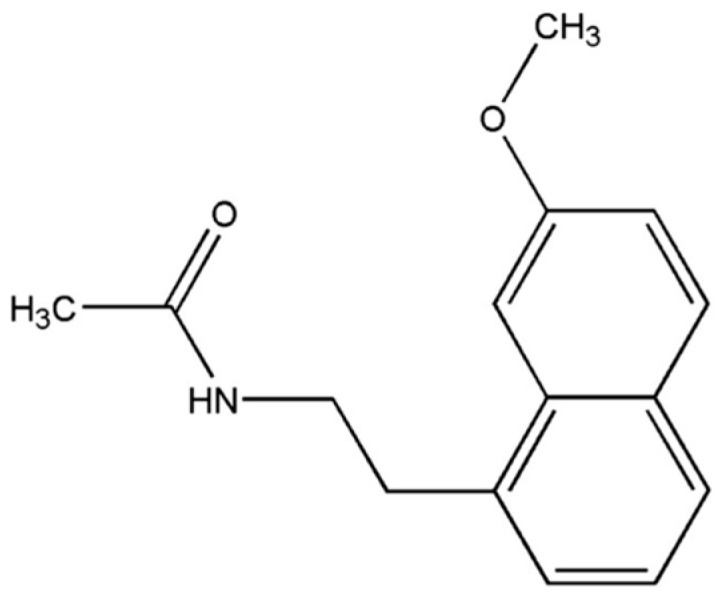
The chemical structure of agomelatine.

In this systematic review, we summarized the evidence on the efficacy and safety of agomelatine (Figure 9) in managing depression with comorbid diabetes, as well as exploring its potential mechanism of action in both diseases.

Several lines of evidence support the efficacy of agomelatine in the management of depression and anxiety symptoms in patients with type 2 diabetes mellitus (T2DM). Agomelatine proves to be at least as effective as SSRIs in reducing depressive symptoms [47,48,49]. Furthermore, agomelatine may be more effective than SSRIs in reducing anxiety symptoms; however, this was applicable only after a longer (12 weeks) period of treatment [30,41,42,50]. What turns out to be most interesting, however, is the effect of agomelatine not on depressive or anxiety symptoms (because its efficacy in this aspect has long been well documented) but on diabetic parameters. Agomelatine significantly decreased HBA1C in patients with diabetes during 12 weeks of therapy [30,41,42], more than SSRIs (namely fluoxetine, sertraline, and paroxetine). Furthermore, agomelatine has no negative effect on metabolic parameters, including liver enzymes, body weight, and lipid profile [36,41,42]. These results suggest that agomelatine has a positive effect on diabetic parameters in the treatment of patients with depression and T2DM and may be a better option than SSRIs in this aspect. The antidiabetic properties of agomelatine may be explained by its melatoninergic effects. Although studies on the antidiabetic effects of agomelatine are scarce, there is ample evidence of a close interaction between melatonin and glucose homeostasis [51]. A recent meta-analysis showed a beneficial effect of melatonin supplementation in patients with diabetes on fasting blood glucose, HBA1C, or insulin resistance [27]. It was also associated with a reduction in markers of inflammation and oxidative stress [52]. As a melatoninergic receptor agonist, agomelatine may affect glucose homeostasis in a way that is at least partly similar to the action of melatonin. Studies have shown that acting on melatoninergic receptors increases SIRT1 expression levels [53,54,55]. SIRT1 is a very important gene involved in glucose homeostasis. It positively affects both insulin levels and insulin sensitivity [56], as well as glucose levels and diabetes symptoms [57]. Agomelatine may reduce inflammation by raising the expression of SIRT1. In animal models, agomelatine was found to reduce blood glucose levels and increase insulin levels through SIRT1 activation, which in turn led to reduced inflammation [58]. In a study by Ozcan et al., it was shown that agomelatine counteracted the formation of diabetes in rats, probably due to its antioxidant effects [36]. This is consistent with previous results that agomelatine administration increased superoxide dismutase (SOD) and catalase (CAT) activity in the posterior cortex and striatum in rats [59]. Therefore, given that antioxidants can protect neurons from oxidative stress-induced damage, the increase in total antioxidant capacity after agomelatine administration may explain its beneficial effects on diabetes prevention [36].

Diabetes is a systemic disease that often results in significant complications, including pain, neuropathy, nephropathy, retinopathy, and cognitive impairment. The treatment of these complications represents a major challenge for contemporary medicine. However, there is emerging evidence that agomelatine may offer a promising solution. Preclinical studies have demonstrated the potential benefits of agomelatine in the context of neuropathy [29,34,37], two in encephalopathy [35,36], and one each in nephropathy [39] and reproductive system effects [38]. In the context of diabetes neuropathy, the serotonergic system is known to play an important role in pain modulation [60,61]. Reports on the role of 5-HT2C receptors in pain are conflicting [62,63,64,65]; however, in general, they show pronociceptive effects, that is, they increase pain sensation. Agomelatine blocks 5-HT2C receptors, thereby further increasing the release of norepinephrine in the limbic system [66,67]. The pathway of action of agomelatine on neuropathic pain may be then an adrenergic transmission through alpha2-adrenergic receptors [34]. Given that adrenergic receptors are involved in the progression of diabetic neuropathy [68,69,70,71] and that noradrenergic system involvement in pain modulation has been described [72,73], agomelatine may act on neuropathic pain, possibly through serotoninergic and alpha2-adrenergic receptors. It has also been found that stimulation of melatoninergic receptors leads to analgesic effects [74,75,76]. The pathway is probably important in agomelatine action, as melatonin shows significant nerve regeneration capacity, neuroprotective potential, and analgesic effects [71,77,78,79,80]. Various studies point to the involvement of MT2 melatoninergic receptors, as well as opioidergic mechanisms in the analgesic effects of melatonin [81,82,83].

The beneficial effect of agomelatine has also been found in a rodent model of encephalopathy and cognitive impairment caused by diabetes. Diabetes severely damages hippocampal synaptic plasticity through various pathological mechanisms [84,85,86]. Studies have shown that agomelatine improves stress-induced cognitive dysfunction in mice, likely through mechanisms involving BDNF signaling, synaptic plasticity, and epigenetic remodeling [87]. In addition, agomelatine may impact genes associated with hippocampal neuroplasticity, such as activity-regulated cytoskeleton-associated protein (Arc), B-cell lymphoma 2 (Bcl2), BDNF, glial cell line-derived neurotrophic factor (GDNF), insulin-like growth factor 1 (IGF1), and neurogenic differentiation factor 1 (NEUROD1) [88]. Agomelatine treatment reduced the number of hippocampal apoptotic cells [89]. The beneficial effects of agomelatine on neuroplasticity have been observed mainly in the prefrontal cortex and hippocampus [90]. In addition, agomelatine may have anti-inflammatory effects via a reduction in IL-1b [36]. The anti-inflammatory properties may also be responsible for the antidepressant effect. Since neural damage is associated with inflammation in the course of diabetes [91], elevated IL-1b levels in the brain may be one of the factors contributing to depression [92]. Diabetes can lead to testicular dysfunction through fibrosis, a complication of various types of inflammation [93], and a significant increase in apoptotic germ cell death, especially in spermatogonia and spermatocytes [94]. The most important diabetes-induced damages in the male reproductive system in diabetes in rat models are inflammatory changes and fibrosis [95,96]. Findings from recent studies suggest that agomelatine may possess anti-apoptotic properties [38]. Activation of pro-inflammatory genes has been postulated [97,98]. Yigitturk et al. showed that AGO decreased the TNFa levels and thus NF-kB, preventing inflammation so that apoptosis and fibrosis of the testicular germ cells are suppressed [38]. It is hypothesized that the prevention of fibrosis may be through blocking 5HT2C receptors [66].

Another frequent complication of diabetes occurring in 30–40% of patients with diabetes is nephropathy. In 1/3 of cases, it leads to renal failure [99]. Studies in animal models have shown that agomelatine raises SIRT1 expression in rat kidneys, which in turn leads to reduced inflammation [39,100]. The anti-inflammatory effects of SIRT1 have been linked to the inhibition of NFkB, which is very important in the changes in diabetic nephropathy [101].

**Figure 10:**
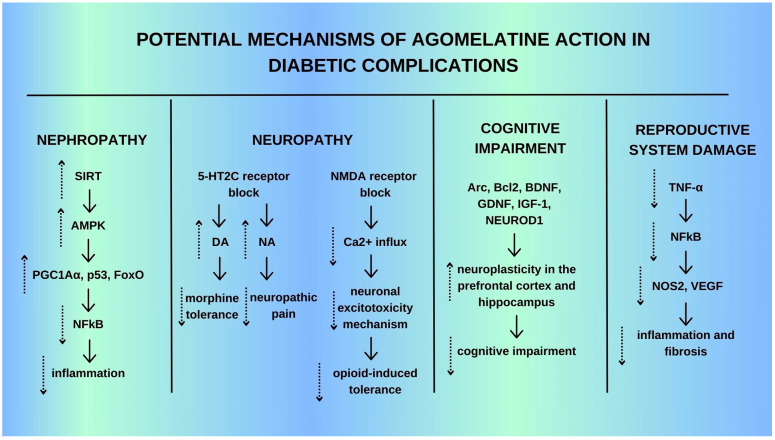
Potential mechanism of agomelatine action in diabetic complications.

The results suggest that agomelatine may provide a range of benefits in diverse conditions through the activation of various neurotransmitter or molecular pathways, both within the central nervous system (CNS) and in specific tissues (Figure 10).

The above mechanisms described in animal models may indicate a potential mechanism of action for agomelatine, both in directly improving the parameters of diabetes control and in preventing distant complications of the disease. Beyond the purely biological mechanism by which agomelatine could improve diabetes control, the behavioral component should not be overlooked. Agomelatine is an effective antidepressant that not only improves mood and activity but also makes it easier for patients to take care of themselves and maintain a healthy lifestyle. One of the studies included in this review evaluated the effect of treatment on the parameters of self-care in patients with diabetes. This is a crucial component of the long-term management of this chronic disease [30]. Interestingly, agomelatine appeared to be better than other antidepressants like SSRIs. Patients in the agomelatine group have higher self-care scores as compared to the SSRI groups.

There are important variables in the context of agomelatine treatment that should be further analyzed. Current studies do not provide enough data to evaluate the efficacy and safety of agomelatine treatment in some subpopulations. The important factor is, for example, age. The secondary study, including one RCT and one open-label study, demonstrated that agomelatine markedly reduced the HDRS and HARS scores in comparison to other SSRIs in patients aged between 50 and 70 years [43]. Furthermore, the study revealed that AGO was more efficacious than paroxetine/fluoxetine in reducing HbA1C levels and BMI in this age group. While additional clinical trials are necessary to substantiate these findings, these results suggest that agomelatine attenuates anxiety and depressive symptoms and enhances diabetic parameters in elderly patients, making it a promising therapeutic option for the future, particularly in this population. Another known confounding factor is, for example, smoking, as it impacts CYP1A2. It seems important to evaluate this and other potentially confounding variables; such studies are lacking.

Finally, an important issue to highlight is the safety of the treatment in patients with diabetes. We conclude that agomelatine is a very safe option for treating depression and anxiety in this population. No study reported significant side effects as a complication of taking this drug. Similarly, no study reported an increase in liver enzymes, which should be monitored during agomelatine treatment. Side effects are very rare and comparable with the group of SSRIs. Some of the common side effects, such as sexual dysfunction or sleep disturbances, are even less frequent on agomelatine treatment than SSRIs. Clinical studies show that agomelatine is a safer treatment than other antidepressants [30,41]. Further clinical studies are needed to assess the safety of agomelatine.

It should be noted that this study is subject to a number of limitations. Firstly, the studies included in the review were based on a relatively small sample size, the populations included were not homogenous, and the clinical characteristics were modest. Secondly, some studies did not include a detailed description of how the participants’ diabetes was treated. In fact, only one study reported continuous diabetes treatment during the study. Thirdly, only one study was an RCT, one was single-blind, and two were open-label studies. Fourthly, we were unable to obtain the raw data from Che et al.’s study [41]. We used the dataset from another published meta-analysis that included the raw data from this study [44,102]. The data from this dataset were compared to other articles that reported raw data, and no misreporting was found, so this dataset was considered a reliable source.

## 5. Conclusions

Agomelatine may be a good choice for patients with diabetes and comorbid depression and anxiety. In addition to improving depressive and anxiety symptoms, it is also beneficial in glycemic control. It has a beneficial effect, better than some SSRIs, on the level of HbA1C, considered so far to be a better option in the population of patients with diabetes. The safety of such treatment is similar to SSRIs; no severe adverse events were reported, and the incidence of some side effects, such as insomnia and sexual dysfunction, are even less often reported. Particularly promising is also its potential action in improving some diabetic complications reported in preclinical trials. However, further clinical studies on larger sample sizes, as well as elucidating its mechanisms of action, especially in the context of diabetic complications, are needed. Also, the variability in its effects on glycemic control highlights the need for more targeted studies. Future research should also focus on identifying the patient subpopulations most likely to benefit from agomelatine treatment.

## Figures and Tables

**Table 1:** Adapted PICO model for preclinical, observational, and interventional trials.

	Preclinical Studies	Observational Studies	Interventional Studies
Patients/ Subjects	Animal models of diabetes.	Patients receiving agomelatine with or without a diabetes diagnosis, under 65 and over 18 years of age.	Patients diagnosed with diabetes under 65 or over 18 years of age.
Intervention	Use agomelatine alone or as add-on therapy.	Patients receiving agomelatine.	Patients treated with agomelatine.
Comparison	No use of agomelatine.	Not receiving agomelatine.	Placebo or other antidepressants.
Outcome	Impact on diabetic parameters or complications of the disease.	Diagnosis of diabetes.	Impact on diabetic parameters or complications of the disease; impact on depressive and anxiety symptoms in patients with diabetes; safety.

## Data Availability

The data presented in this study are available upon request from the corresponding author. The data are not publicly available due to privacy or ethical concerns.

## Supplementary Materials

The following supporting information can be downloaded at https://www.mdpi.com/article/10.3390/ijms252312631/s1.
