# Supplementary material for: Efficacy and Safety of Agomelatine in Depressed Patients with Diabetes: A Systematic Review and Meta-Analysis
Source: Int J Mol Sci. 2024 Nov 25;25(23):12631. doi: 10.3390/ijms252312631 (PMC11641584; doi:10.3390/ijms252312631)
Supplement: Supplementary file 1 [file ijms-25-12631-s001.zip › ijms-3316093-supplementary.pdf]

International Journal of
Molecular Sciences

MDPI

Table S1. Studies using animal models.

Author, year, country	Species, Sample size (N), Sex (male/female), Diabetes model	Drugs Tested	Procedures/ Measurments	Results: 1) diabetic parameters; 2) diabetic complications; 3) markers/mechanism of AGO action
Aydin et al. 2016 [29] Turkey	Sprague Dawley rats N=8/group Sex - no information STZ induced diabetes model; neuropathy	Agomelatine 40, 80 mg/kg Pregabalin 10 mg/kg 14 days of treatment	FBG, OGTT, Metabolic cage measurements, Activity cage test, Randall–Selitto test , Dynamic plantar test, Hargreave's test (plantar test), Cold (4 °C) plate test, Warm (38 °C) plate test Mechanistic studies after PCPA, AMPT, phentolamine and propranolol administration	1) Administration of AGO had no significant effect on diabetic (FBG, OGTT) and metabolic parameters (water and feed consumption, urine and fecal excretion), body weight, and locomotor activity.<br>2) AGO significantly improved hyperalgesia and allodynia responses (mechanical and thermal). A significant interaction was found between treatment and time.<br>3) PCPA and AMPT pretreatments indicated that the observed effect of agomelatine was related to catecholamine level. Phentolamine and propranolol administration point to both types of adrenoceptors (alfa and beta) contribution to the pharmacological action of AGO on neuropathic pain.

Mahmoud et al. 2023 [39] Egypt	Wistar rats N=5-6/group Sex - males only Fructose and STZ induced diabetes model; nephropathy	Agomelatine 20 mg/kg Agomelatine 20 mg/kg + Luzindole 5 mg/kg Agomelatine 20 mg/kg + EX527 5 mg/kg 6 weeks of treatment	Histological examination, Immunohistochemical analysis (SIRT1 expression), FBG and serum insulin analysis, HOMA-IR, Kidney function tests, phospho- and total NFκB, AMPK, ICAM-1, VCAM-1, MCP-1, SIRT1 mRNA expression	1) AGO administration decreased FBG, HOMA-IR and caused higher serum insulin levels. 2) AGO administration improved kidney morphology (indicated by a marked reduction in interstitial fibrosis, tubular epithelial degeneration) and function (serum creatinine, urea, KIM-1). 3) AGO treatment increased SIRT1 expression, reduced renal NFκB phosphorylation, increased renal AMPK phosphorylation and improved renal inflammation (reduced level of ICAM-1, VCAM-1, MCP-1). Treatment with either luzindole (SIRT1 inhibitor) or EX527 (melatonin receptor inhibitor) abrogated the effects of AGO.
Ozcan et al. 2019 [36] Turkey	Balb/C mice N=7/group Sex - male only STZ induced diabetes model, encephalopathy	Agomelatine pretreatment 5, 10, 20 mg/kg Agomelatine treatment twice daily 10 mg/kg for 15 days	Plasma insulin, FBG and total antioxidant capacity, triglyceride, total cholesterol, HDL and LDL levels, RNA isolation and synthesis gene expression analysis with RT-PCR (brain regions: raphe nucleus, PAG, amygdala, and nucleus accumbens)	1) Pretreatment of AGO (5 mg/kg,10 mg/kg, 20 mg/kg) caused a decrease in FBG and increased plasma insulin level. These effects were dose-dependent. AGO pretreatment did not affect triglyceride, total cholesterol, HDL, and LDL levels. TAC levels were significantly higher in 10 mg/kg agomelatine groups compared to healthy and diabetic control. 2) AGO reversed high glucose–induced cell viability decreases in DRG neurons. 3) AGO treatment increased total antioxidant capacity. AGO acute and chronic treatment decreased IL-1β mRNA levels in only nucleus accumbens and raphe nucleus. Chronic AGO treatment decreased TACR1 mRNA levels in raphe nucleus, PAG, amygdala, and nucleus accumbens. Acute AGO treatment decreased TACR1 mRNA levels in raphe nucleus, PAG, and amygdala except nucleus accumbens.
Chenaf et al. 2017 [34] France	Sprague-Dawley rats N=6-8/group Sex - males only STZ induced diabetes model, neuropathy	Agomelatine 10, 20 and 45 mg/kg, Gabapentin 50 mg/kg	Paw pressure test, Rotarod test	1) No information. 2) AGO reduced hypersensitivity in diabetic rats. For the doses of 20 and 45 mg/kg the reversal was complete. The effect of AGO was higher than gabapentin. There was no significant decrease in fall latencies, after administration of AGO 45 mg/kg, gabapentin 50 mg/kg or their combination.

				3) The anti-hypersensitivity effect of agomelatine involved melatonergic, 5-HT2C and alfa-2 adrenergic receptors but not beta adrenoceptors.
Can et al. 2018 [35] Turkey	Sprague-Dawley rats N=6-8/group Sex -males only STZ induced diabetes model, cognitive impairment	Agomelatine 40, 80 mg/kg Piracetam 200 mg/kg	Morris Water Maze Test, Passive Avoidance Test, Activity Cage Test, Rota-rod Test, Optical Fractionator Counting Method	1) No information.<br>2) AGO treatment effectively reversed the impaired learning, memory performance, and emotional learning of diabetic rats with comparable efficacy to piracetam. AGO caused no significant change either in the total number of locomotor activities or in the falling latencies of diabetic animals.<br>3) AGO treatment reversed diabetes-induced reduction of cells in the CA1–3 regions and decrease in the dentate gyrus.
Ozcan et al. 2020 [37] Turkey	Balb/C mice N=7/group Sex - males only STZ induced diabetes model, neuropathy	Agomelatine 10 mg/kg, Morphine 10 mg/kg, Agomelatine + Morphine both 10mg/kg<br><br>Acute treatment: morphine 0.1, 1, 5 and 10 mg/kg; agomelatine 0.1, 1, 5 and 10 mg/kg	Hot plate test, GluN1 and GAPDH in raphe nucleus and PAG	1) No information.<br>2) Acute treatment of AGO (10 mg/kg) increased the pain threshold. After combination with morphine analgesic threshold values were higher. AGO augmented the peak of morphine and shifted the time of its peak earlier. Chronic treatment of AGO and AGO with morphine was sustained for the duration of treatment contrary to morphine alone.<br>3) AGO co-administration with morphine limited the increase in GluN1 mRNA levels in raphe nucleus and significantly attenuated the increase in GluN1 mRNA levels in PAG then morphine alone.

Yigitturk et al. 2017 [38] Turkey	Sprague Dawley albino rats<br>N=7/group<br>Sex - males only<br>STZ induced diabetes model, testicular damage	Agomelatine 20 mg/kg<br>Gallic acid 20 mg/kg	Blood glucose level, evaluation of lipid peroxidation MDA levels with TBARS, determination of tissue SOD and catalase activity, histopathological examination, TNFα, NOS2, fibronectin and VEGF immunoexpressions, TUNEL analysis	1) No beneficial effect on the blood glucose level was observed.<br>2) The loss of somatic sertoli cells and spermatogenic series cells was significantly decreased in the AGO group, compared to the diabetes without treatment. No atypical cells were seen in the lumen.<br>3) The MDA levels were significantly decreased in the AGO compared to diabetic rats without treatment. CAT and SOD enzyme levels were significantly increased. TNFα, NOS2, fibronectin, VEGF expressions, and TUNEL positive cell were significantly decreased in the AGO group, compared to the diabetes without treatment.

FBG - Fasting blood glucose, AG - Agomelatine, PAG - Periaqueductal Gyrus, OGTT - Oral Glucose Tolerance, Test, TAC - Total Antioxidant Capacity, MDA - Malondialdehyde, TUNEL - Terminal Deoxynucleotidyl, Transferase-Mediated Deoxyuridine Triphosphate Nick End Labeling, HOMA-IR - Homeostasis Model Assessment – Insulin Resistance, PCPA - p-Chlorophenylalanine Methyl Ester, AMPT - α-Methyl-Para-Tyrosine Methyl Ester, AMPK - AMP - activated protein kinase, TACR1 - Tachykinin Receptor 1, ICAM-1 - Intercellular Adhesion Molecule 1, VCAM-1 - Vascular Cell Adhesion Molecule 1, MCP-1 – Monocyte Chemoattractant Protein 1, IL-1β - Interleukin-1 beta, GluN1 - glutamate ionotropic receptor N-methyl-D-aspartate, TNFα - Tumor Necrosis Factor α, NOS2 - Nitric Oxide Synthase 2, VEGF - Vascular Endothelial Growth Factor, TBARS - Thiobarbituric Acid Reactive Substance, SOD - Superoxide Dismutase, HDL - High-Density Lipoprotein, LDL - Low-Density Lipoprotein, GAPDH - Glyceraldehyde-3-Phosphate Dehydrogenase, mRNA - messenger RNA
